# Blockade of P2X4 Receptors Inhibits Neuropathic Pain-Related Behavior by Preventing MMP-9 Activation and, Consequently, Pronociceptive Interleukin Release in a Rat Model

**DOI:** 10.3389/fphar.2017.00048

**Published:** 2017-02-16

**Authors:** Agnieszka M. Jurga, Anna Piotrowska, Wioletta Makuch, Barbara Przewlocka, Joanna Mika

**Affiliations:** Institute of Pharmacology, Polish Academy of Sciences, Department of Pain PharmacologyKrakow, Poland

**Keywords:** antinociceptive interleukins, buprenorphine, chronic constriction injury, CORM-2, morphine, neuropathy, p38MAPK, PI3K/Akt

## Abstract

Neuropathic pain is still an extremely important problem in today’s medicine because opioids, which are commonly used to reduce pain, have limited efficacy in this type of pathology. Therefore, complementary therapy is needed. Our experiments were performed in rats to evaluate the contribution of the purinergic system, especially P2X4 receptor (P2X4R), in the modulation of glia activation and, consequently, the levels of nociceptive interleukins after chronic constriction injury (CCI) of the right sciatic nerve, a rat model of neuropathic pain. Moreover, we studied how intrathecal (*ith.*) injection of a P2X4R antagonist Tricarbonyldichlororuthenium (II) dimer (CORM-2) modulates nociceptive transmission and opioid effectiveness in the CCI model. Our results demonstrate that repeated *ith.* administration of CORM-2 once daily (20 μg/5 μl, 16 and 1 h before CCI and then daily) for eight consecutive days significantly reduced pain-related behavior and activation of both spinal microglia and/or astroglia induced by CCI. Moreover, even a single administration of CORM-2 on day 7 after CCI attenuated mechanical and thermal hypersensitivity as efficiently as morphine and buprenorphine. In addition, using Western blot, we have shown that repeated *ith.* administration of CORM-2 lowers the CCI-elevated level of MMP-9 and pronociceptive interleukins (IL-1β, IL-18, IL-6) in the dorsal L4-L6 spinal cord and/or DRG. Furthermore, in parallel, CORM-2 upregulates spinal IL-1Ra; however, it does not influence other antinociceptive factors, IL-10 and IL-18BP. Additionally, based on our biochemical results, we hypothesize that p38MAPK, ERK1/2 and PI3K/Akt but not the NLRP3/Caspase-1 pathway are partly involved in the CORM-2 analgesic effects in rat neuropathic pain. Our data provide new evidence that P2X4R may indeed play a significant role in neuropathic pain development by modulating neuroimmune interactions in the spinal cord and DRG, suggesting that its blockade may have potential therapeutic utility.

## Introduction

The neuropathic pain state, which can occur after injury of a nerve or be a concurrent symptom of various clinical disorders, is considered to be one of the most important problems in medicine today (Burma et al., 2016; Burke et al., 2017). The limited understanding of its pathomechanism prevents the development of an effective treatment. Opioid drugs, used commonly in clinics for inflammatory pain therapy, lose their efficacy in neuropathic pain (Furlan et al., 2006). In recent years, a rising number of reports have confirmed the crucial role of microglial cells in neuropathic pain development (Tsuda et al., 2003; Beggs and Salter, 2013). Those cells have been shown to be strongly activated after peripheral nerve injury on the ipsilateral side of the spinal cord (Tsuda et al., 2003; Beggs and Salter, 2013). This is directly connected with the morphological changes of microglia into an amoeboid form and the enhanced expression of nociceptive factors and microglial receptors levels (Beggs and Salter, 2013). Matrix metalloproteinase 9 (MMP-9) appears to induce the development of neuropathic pain by active cleavage of IL-1β and activating p38MAPK in spinal microglia (Schonbeck et al., 1998; Kawasaki et al., 2008). While activated, microglial cells are known to release pronociceptive cytokines (for instance IL-1β, IL-6, and IL-18), which cause pain and counteract opioid analgesia (DeLeo and Yezierski, 2001; Mika et al., 2008; Narita et al., 2008). Recently, many studies have highlighted the crucial role of antinociceptive cytokines (IL-1Ra, IL-10, and IL-18BP) against pain (Ledeboer et al., 2007; Mika et al., 2013; Pilat et al., 2015, 2016). We, as well as other laboratories, have previously reported that inhibiting microglia activation with minocycline significantly reduces pain behavior and the level of pronociceptive interleukins and enhances the effectiveness of morphine in neuropathy (Mika et al., 2007; Cui et al., 2008; Zychowska et al., 2016).

Certain subtypes of purinergic ionotropic (P2X) receptors are reported to contribute to neuropathic pain development (Tsuda et al., 2003; Grygorowicz et al., 2016). We decided to take a close look at microglial subtype 4 (P2X4R) because of the increasing number of studies indicating its importance in neuropathy pathogenesis when activated by its endogenous agonist, adenosine, 5′-triphosphate (ATP) (Sim and North, 2010). Elevated levels of this nucleotide are observed after injuries, which induce the depolarization of sensory neurons (North and Khakh, 2006). The role of ATP was examined in various contexts, e.g., in knock-out mouse models or pharmacomodulation (Cockayne et al., 2000; Tsuda et al., 2003). Ulmann et al. (2008) examined P2X4 knockout mice, which showed a remarkable reduction in mechanical hypersensitivity after spinal nerve injury in comparison with wild-type animals. P2X4R might thus represent a potential therapeutic target to limit microglia-mediated inflammatory responses associated with nervous system injury (Ulmann et al., 2008). There is abundant evidence that extracellular ATP plays an important role in pain signaling in both the peripheral and central nervous system. It has been demonstrated that extracellular ATP and/or its metabolites are factors possibly involved in the transduction of mechanical stimuli into enhanced excitability of nociceptive nerve fibers in the trunk of a peripheral nerve, and it is well-documented that ATP is released by different types of mechanical stimuli (Irnich et al., 2002; Lazarowski et al., 2003). Gemes et al. (2012) indicated that nerve injury increases plasma membrane Ca^2+^-ATPase activity in sensory neurons after spinal nerve ligation. It has also been shown that an antagonist of all P2X receptors subtypes (TNP-ATP) but not an antagonist specific to all subtypes excluding P2X4R (PPADS) has a strong analgesic potency and ability to block P2X4R upregulation on microglial cell cultures, pointing to the crucial role of this specific P2X receptor subtype in pain sensation (Tsuda et al., 2003). Tricarbonyldichlororuthenium (II) dimer (CORM-2) has been reported to be an effective P2X4R antagonist (Wilkinson and Kemp, 2011). We have also recently reported that repeated intraperitoneal (*ip.*) administration of CORM-2 significantly reduced mechanical and thermal hypersensitivity in a rat model of neuropathy, and as preliminary data, that repeated intrathecal (*ith.*) administration attenuate thermal hypersensitivity. Moreover, a single *ip.* administration of CORM-2 was able to enhance opioid analgesia (Jurga et al., 2016a).

Despite the many studies on the topic, the involvement and changes of the P2X4R under neuropathic pain remain to be elucidated, but the P2X4R is known to be localized mainly on microglia/macrophage cells. Therefore, we started our experiments with an evaluation of the time course changes in P2X4R levels in a rat neuropathic pain model [chronic constriction injury of the sciatic nerve (CCI)]. Next, we decided to use behavioral tests to examine if or how CORM-2 injected intrathecally (*ith.*) can influence nociceptive transmission under neuropathic pain. Moreover, using the Western blot technique, we have measured the influence of CORM-2 on activated glia and, in parallel, analyzed the protein levels of both the pronociceptive (IL-1β, IL-18, IL-6) and antinociceptive (IL-1Ra, IL-18BP, IL-10) factors in the dorsal horn of the lumbar spinal cord and DRG in the CCI-exposed rats. Knowing that the mature forms of IL-1β and IL-18 are cleaved by MMP-9 (Schonbeck et al., 1998; Kawasaki et al., 2008) and/or nucleotide-binding oligomerization domain, leucine rich repeat and pyrin domain containing 3/caspase 1 (NLRP3/Casp-1) inflammasome complex (Franchi et al., 2009), we decided to examine the influence of CORM-2 on the activation of those factors in the neuropathic pain model. Additionally, we investigated the impact of this P2X4R antagonist on the activation of the p38MAPK and ERK1/2 pathways and changes in NFκB activation, which are known to be crucial for the production of nociceptive factors during neuropathic pain development (Tsuda et al., 2004; Hains and Waxman, 2006). Recent publications report also contribution of phosphoinositide 3-kinase PI3K/Akt pathway in development of hypersensitivity in different pain models (Tsuda et al., 2009; Pereira et al., 2011; Xu et al., 2014), so we have included it in our experiments. Moreover, we investigated how a single intrathecal administration of CORM-2 would influence opioid (morphine and buprenorphine) analgesia in a rat model of neuropathic pain.

## Materials and Methods

### Animals

Male Wistar rats (250–300 g) from Charles River (Hamburg, Germany) were housed in cages lined with sawdust under a standard 12/12 h light/dark cycle (lights on at 06:00 a.m.) with food and water available *ad libitum*. Only the minimal essential number of animals was used, and all of the procedures were performed according to the recommendations of IASP (Zimmermann, 1983) and the NIH Guide for the Care and Use of Laboratory Animals and this study was carried out in accordance with the recommendations of local Ethics Committee (Krakow, Poland).

### Catheter Implantation

The intrathecal (*ith.*) injections were achieved through implanting catheters according to the method of Yaksh and Rudy (Yaksh and Rudy, 1976) under pentobarbital (60 mg/kg; *ip.*) anesthesia, as was reported previously (Popiolek-Barczyk et al., 2014). After implantation, the animals were allowed to recover for a minimum of 1 week before the experiment.

### Chronic Constriction Injury (CCI)

Chronic Constriction Injury was performed according to Bennett and Xie (1988) under sodium pentobarbital anesthesia (60 mg/kg; *ip.*). The procedure was described in detail in our previous paper (Jurga et al., 2016a). As predicted, the CCI surgery caused long-lasting neuropathic pain behaviors, such as mechanical and thermal hypersensitivity, in all of the operated rats.

### Drug Administration

CORM-2 [*tricarbonyldichlororuthenium (II) dimer*, Sigma-Aldrich, St. Louis, MO, USA] was administered repeatedly once daily (20 μg/5 μl, *ith.*; dissolved in water for injection) for 9 consecutive days. CORM-2 were delivered by producer in sealed vial, which was opened shortly before experiments, and then dissolved in freshly opened sterile water for injections in autoclaved probe. The CCI surgery was defined as day 0; CORM-2 was administered 16 h (day -1, late afternoon) and 1 h (day 0, early morning) before CCI and then daily from day 1 to 7 after CCI (*Scheme on* **Figure 2A**). This method of CORM-2 or vehicle administration was used throughout the work and is referred to as “repeated administration” in the text.

Morphine (2.5 μg/5 μl, *ith.*) or buprenorphine (2.5 μg/5 μl, *ith.*) was administered as a single injection 2 h after CORM-2 on day 7 after CCI, and the behavioral tests were conducted 30 min later. The control groups received vehicle (water for injection) according to the same schedule (**Figure 8A**).

### Behavioral Tests

Two behavioral tests, von Frey’s and the cold plate, were performed on day 7 after CCI, 2 h after the drug or vehicle dose. The control groups received vehicle (water for injection) according to the same schedule (**Figure 2A**). During the behavioral tests on day 7, person responsible for substance administration were passing animals from cages directly to the von Frey apparatus cage, and then to the cold plate box, where two different experimentators were performing tests.

#### Von Frey’s Test

Mechanical hypersensitivity was measured in the rats subjected to CCI using an automatic von Frey apparatus (Dynamic Plantar Aesthesiometer, Ugo Basile, Italy). Rats were placed in plastic cages with a wire net floor, and a von Frey’s filament was applied to the midplantar surface of the CCI-exposed hind foot. Measurements were taken automatically with a cut-off at 26 g (Makuch et al., 2013).

#### Cold Plate Test

Thermal hypersensitivity was assessed using the Cold/Hot Plate Analgesia Meter (Columbus Instruments, USA) as described previously (Mika et al., 2007). The temperature of the cold plate was maintained at 5°C, and the cut-off latency of a single measurement was 30 s. The rats were placed on the cold plate, and the time until the hind foot was lifted was recorded. The injured foot was the first to react in every case.

### Western Blot Analysis

Rat ipsilateral dorsal lumbar (L4-L6) spinal cords and DRG were collected immediately after decapitation on day 7 after CCI, 6 h after the last drug administration. Tissue was stored at -80°C until processing, as described previously (Rojewska et al., 2014). Blots were incubated overnight at 4°C with primary antibodies against the following proteins: IBA-1 (rabbit anti-rat, 1:1000, Proteintech), GFAP (rabbit anti-rat, 1:10 000, Novus), P2X4R (rabbit anti-rat, 1:500, Alomone), IL-1β (rabbit anti-rat, 1:1000, Abcam), IL-18 (rabbit anti-rat, 1:1000, Abcam), IL-6 (rabbit anti-rat, 1:500, Invitrogen), MMP-9 (rabbit anti-rat, 1:1000, EMD Millipore), TIMP-1 (rabbit anti-rat, 1:1000, Novus biologicals), IL-1Ra (rabbit anti-rat, 1:1000, Abcam), IL-18BP (rabbit anti-rat, 1:1000, Novus biologicals), IL-10 (rabbit anti-rat, 1:1000, Invitrogen), p38MAPK (rabbit anti-rat, 1:1000, Cell Signaling), p-p38 MAPK (rabbit anti-rat, 1:200, Cell Signaling), ERK1/2 (rabbit anti-rat, 1:500, Cell Signaling), p-ERK1/2 (rabbit anti-rat, 1:500, Cell Signaling), NFκB (rabbit anti-rat, 1:1000, Santa Cruz), p-NFκB (rabbit anti-rat, 1:1000, Santa Cruz), PI3K (rabbit anti-rat, 1:1000, Cell Signaling), Akt (rabbit anti-rat, 1:1000, Cell Signaling), p-Akt (rabbit anti-rat, 1:1000, Cell Signaling), NLRP3 (rabbit anti-rat, 1:500, Santa Cruz), Caspase-1 (rabbit anti-rat, 1:500, Abcam), and GAPDH (mouse anti-rat, 1:5000, Merck Millipore) as a loading control. Blots were incubated for 1 h at RT with a corresponding secondary polyclonal antibody conjugated to HRP (goat anti-rabbit IgG or horse anti-mouse, 1:5000, Vector laboratories). Antibodies were diluted in a *Signal Boost Immunoreaction Enhancer Kit* (Merck Millipore). Immunocomplexes were detected using the *Clarity^TM^ Western ECL Substrate* (BioRad) and visualized using a Fujifilm LAS-4000 fluoroimager system.

### Data Analysis

Since, we have shown (Rojewska et al., 2015) that there are no differences in neither nociceptive responses nor biochemical analysis of protein levels between INTACT (not operated) and SHAM (operated, without nerve injury) rats, we used only INTACT animals for the experiments in the present study. **Behavioral data** are presented as the mean ± SEM of 5–10 rats per group. Tests were performed on the following rat groups: **INTACT**: healthy rats; **V**: vehicle-treated, CCI-exposed; **CORM-2**: CORM-2*-*treated, CCI-exposed; **V+M**: vehicle-treated with morphine administration, CCI-exposed; **V+B**: vehicle-treated with buprenorphine administration, CCI-exposed; **CORM-2+M**: CORM-2-treated with morphine administration, CCI-exposed; and **CORM-2+B**: CORM-2-treated with buprenorphine administration, CCI-exposed. Inter-group differences were statistically evaluated by one-way ANOVA followed by Bonferroni’s *post hoc* test using GraphPad Prism 7 software. Significance was defined as follows: ^∗∗∗^*p <* 0.001 compared to INTACT rats; ^#^*p <* 0.05; ^##^*p <* 0.01; and ^###^*p <* 0.001 compared to V-treated CCI-exposed rats; *p* < 0.01 and *p* < 0.001 compared to CORM-2-treated CCI-exposed rats; ^$^*p* < 0.05 and ^$$$^*p* < 0.001 between the V-CCI-opioid-treated groups and CORM-2-opioid-treated groups. **Protein analyses** were performed using the Western blot method. The analyses of the ipsilateral, dorsal lumbar (L4-L6) spinal cord and DRG were performed in INTACT, V-treated CCI-exposed group, and CORM-2-treated CCI-exposed group. The results are presented as fold changes compared to the INTACT group. The data are presented as the mean ± SEM and represent the normalized averages derived from the analysis of 4–7 samples for each group. The analysis was performed with the FUJIFILM Multi Gage V3.0 analysis program. Inter-group differences were analyzed using one-way ANOVA followed by Bonferroni’s multiple comparisons test using GraphPad Prism 7 software. ^∗^*p <* 0.05; ^∗∗^*p <* 0.01; and ^∗∗∗^*p <* 0.001 indicate a significant difference compared to INTACT rats; and ^#^*p <* 0.05; ^##^*p <* 0.01; and ^###^*p <* 0.001 indicate a significant difference compared to V-treated CCI-exposed rats.

## Results

### Changes in P2X4R, IBA-1 and GFAP Protein Levels in the Spinal Cord and DRG, as Measured on Days 2, 7, and 14 after CCI

**FIGURE 1:**
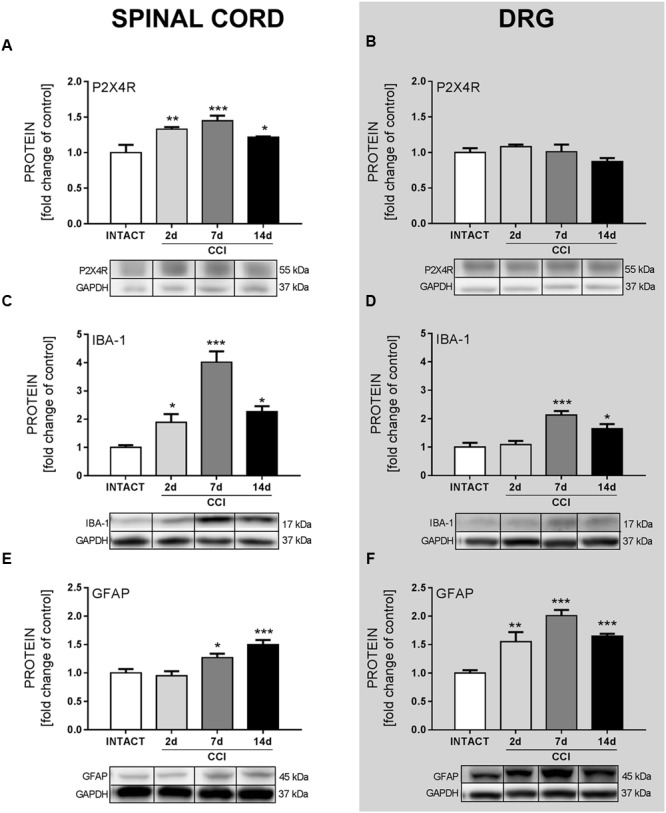
**The Western blot analysis of the protein levels of the P2X4R (A,B)**, IBA-1 **(C,D)**, and GFAP **(E,F)** in the ipsilateral dorsal lumbar spinal cord **(A,C,E)** and dorsal root ganglia (DRG) **(B,D,F)** tissue on days 2, 7, and 14 after chronic constriction injury (CCI) in rats. The representative bands are shown below each column of respective group on the graph and come from the same membrane photo. Samples from different groups were not next to each other so were cut from different locations and set together. Data are presented as the mean ± SEM of 4–7 samples per group. Inter-group differences were analyzed using one-way ANOVA followed by Bonferroni’s multiple comparisons test. ^∗^*p* < 0.05, ^∗∗^*p* < 0.01, and ^∗∗∗^*p* < 0.001 compared to the INTACT group.

Upregulation of P2X4R levels in the spinal cord was significant at every time point (day 2: *p* < 0.01, day 7: *p* < 0.001, day 14: *p* < 0.05, **Figure 1A**), but there was no change in the DRG (**Figure 1B**) after CCI comparing to INTACT group. We have observed, in both the spinal cord and DRG, that IBA-1 protein level was the most elevated on day 7 after CCI (*p* < 0.001, **Figures 1C,D**) and slightly less but still significantly elevated on day 14 after CCI (*p* < 0.05, **Figures 1C,D**) comparing to INTACT group. GFAP started to upregulate in the spinal cord on day 7 after CCI (*p* < 0.05, **Figure 1C**) with the highest level on day 14 (*p* < 0.001, **Figure 1E**); in the DRG, this elevation started on day 2 after CCI (*p* < 0.01, **Figure 1F**) and lasted through days 7 and 14 (both *p* < 0.001, **Figure 1F**) comparing to healthy animals.

### Effects of Chronic Intrathecal CORM-2 Administration on Hypersensitivity, P2X4R, IBA-1, and GFAP Levels on Day 7 after CCI

**FIGURE 2:**
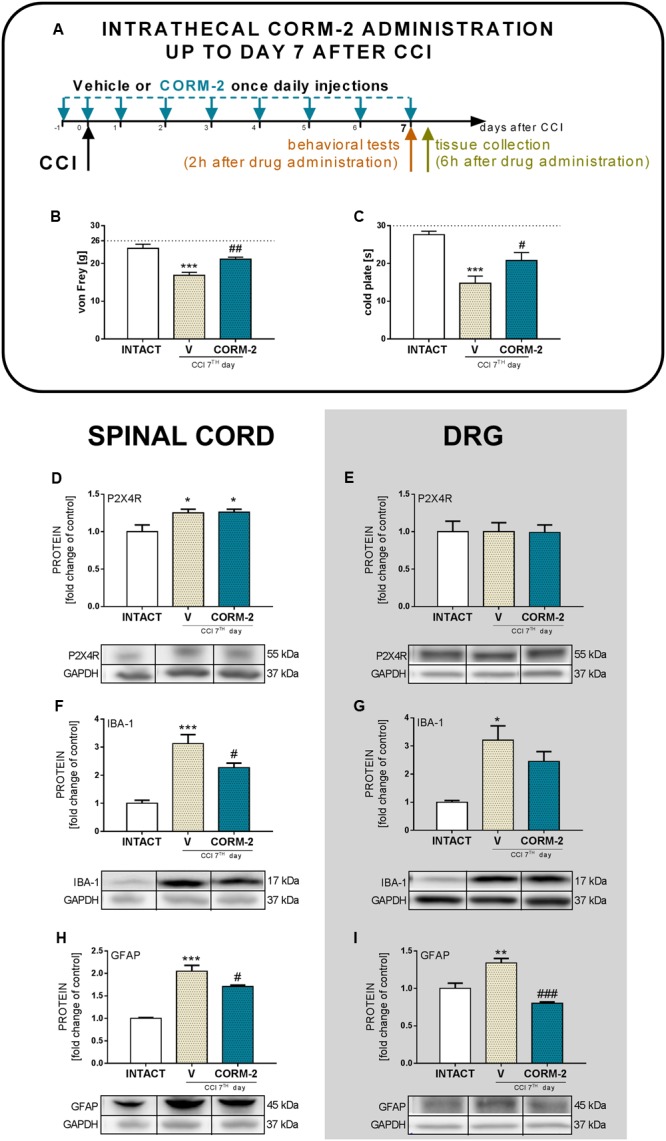
**Effects of repeated *ith.* CORM-2 administration (20 μg/5 μl, *ith.* 16 and 1 h before CCI and then once daily for 7 days) on the development of mechanical** (**B**; von Frey’s test) and thermal (**C**; cold plate test) hypersensitivity on day 7 after CCI as measured 2 h after the last drug administration **(A)**. The behavioral results are presented as the mean ± SEM (7–10 rats per group), and the horizontal dotted line shows the cut-off value (**B**: 26 g, **C**: 30 s). The Western blot analysis **(D–I)** shows the influence of chronic *ith.* CORM-2 administration on protein levels of the P2X4R **(D,E)**, IBA-1 **(F,G)** and GFAP **(H,I)** in the spinal cord **(D,F,H)** and dorsal root ganglia (DRG) **(E,G,I)** on day 7 after CCI 6 h after the last drug administration. The representative bands are shown below each column of respective group on the graph and come from the same membrane photo. Samples from different groups were not next to each other so were cut from different locations and set together. Biochemical results are presented as the mean ± SEM (4–7 rats per group). The inter-group differences were analyzed using one-way ANOVA with Bonferroni’s multiple comparisons test. ^∗^*p* < 0.05, ^∗∗^*p* < 0.01, and ^∗∗∗^*p* < 0.001 compared to the INTACT animals; ^#^*p* < 0.05; ^##^*p* < 0.01; and ^###^*p* < 0.001 compared to the V-treated CCI group.

Chronic constriction injury caused pain-related behavior and repeated administration of CORM-2 (20 μg/5 μl, *ith.*), an antagonist of P2X4R, significantly attenuated mechanical (*p* < 0.01, **Figure 2B**) and thermal (*p* < 0.05, **Figure 2C**) hypersensitivity as measured 2 h after the injection on day 7 (**Figure 2A**). However, CORM-2 did not influence the level of P2X4R in the spinal cord (**Figure 2D**) or DRG (**Figure 2E**) which was respectively: elevated and unchanged comparing to INTACT group. Moreover, we observed that CORM-2 influenced glia activation. CCI caused and elevation of glia markers levels in spinal cord and DRG comparing to INTACT group and CORM-2 diminished activation of IBA-1-positive cells in the spinal cord (*p* < 0.001, **Figure 2F**) but not in the DRG (**Figure 2G**). The protein level of GFAP was elevated in the V-treated CCI-exposed group and was downregulated by CORM-2 in the spinal cord (*p* < 0.05, **Figure 2H**) and DRG (*p* < 0.001, **Figure 2I**).

### Effects of Chronic Intrathecal CORM-2 Administration on Protein Levels of Pronociceptive Interleukins in the Spinal Cord and DRG on Day 7 after CCI

**FIGURE 3:**
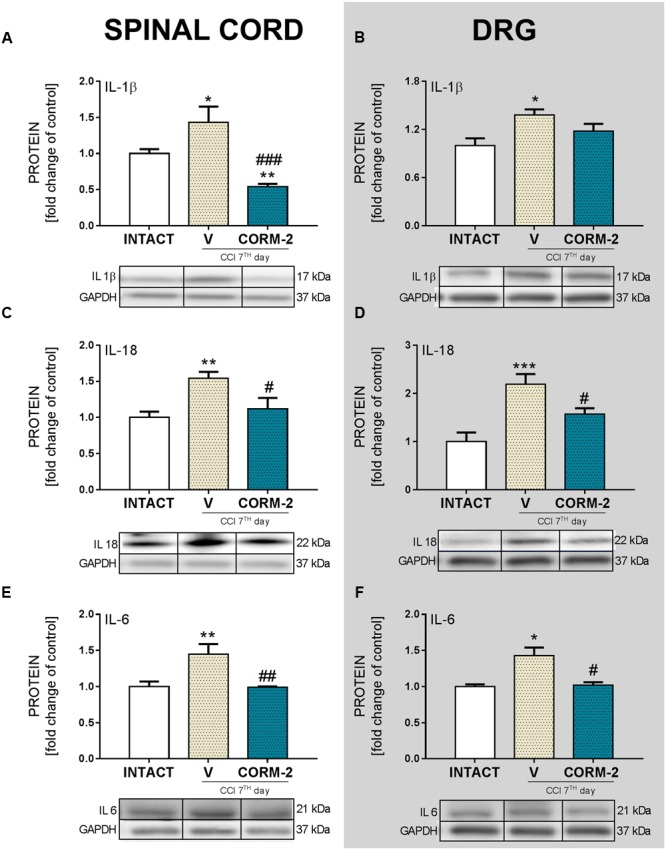
**The Western blot analysis shows the influence of repeated *ith.* CORM-2 administration on protein levels of IL-1β (A,B)**, IL-18 **(C,D)**, and IL-6 **(E,F)** in the ipsilateral dorsal lumbar spinal cord **(A,C,E)** and dorsal root ganglia (DRG) **(B,D,F)** tissue on day 7 after chronic constriction injury (CCI) in rats 6 h after the last drug administration. The representative bands are shown below each column of respective group on the graph and come from the same membrane photo. Samples from different groups were not next to each other so were cut from different locations and set together. Data are presented as the mean ± SEM of 4–7 samples per group. Inter-group differences were analyzed using one-way ANOVA followed by Bonferroni’s multiple comparisons test. ^∗^*p* < 0.05, ^∗∗^*p* < 0.01, and ^∗∗∗^*p* < 0.001 compared to the INTACT group; ^#^*p* < 0.05, ^##^*p* < 0.01; and ^###^*p* < 0.001 compared to the V-treated CCI group.

Repeated CORM-2 administration (20 μg/5 μl, *ith.*) significantly prevented activation of several pronociceptive factors during neuropathic pain. We observed that the protein level of IL-1β was significantly elevated after CCI comparing to INTACT group and lower in the CORM-2-treated group than in the V-treated CCI-exposed group in the spinal cord (*p* < 0.001, **Figure 3A**) but the effect was not significant in the DRG (**Figure 3B**). IL-18 protein levels were elevated after CCI comparing to INTACT group and significantly downregulated after P2X4R antagonist administration in both the spinal cord and DRG (*p* < 0.05, **Figures 3C,D**). CORM-2 also prevented upregulation of IL-6 in the spinal cord (*p* < 0.01, **Figure 3E**) and DRG (*p* < 0.05, **Figure 3F**).

### Effects of Chronic Intrathecal CORM-2 Administration on MMP-9 and TIMP-1 Protein Levels in the Spinal Cord and DRG on Day 7 after CCI

**FIGURE 4:**
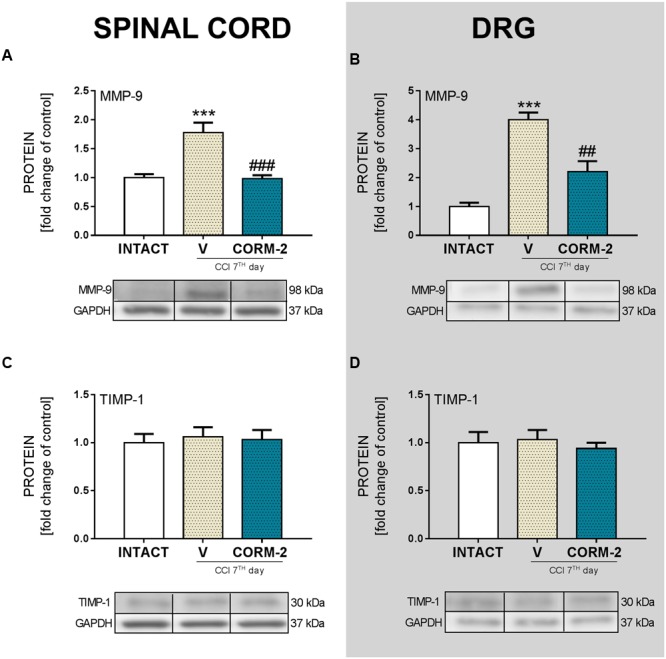
**The Western blot analysis shows the influence of repeated *ith.* CORM-2 administration on protein levels of MMP-9 (A,B)** and TIMP-1 **(C,D)** in the ipsilateral dorsal lumbar spinal cord **(A,C)** and dorsal root ganglia (DRG) **(B,D)** tissue on day 7 after chronic constriction injury (CCI) in rats 6 h after the last drug administration. The representative bands are shown below each column of respective group on the graph and come from the same membrane photo. Samples from different groups were not next to each other so were cut from different locations and set together. Data are presented as the mean ± SEM of 4–7 samples per group. Inter-group differences were analyzed using one-way ANOVA followed by Bonferroni’s multiple comparisons test. ^∗∗∗^*p* < 0.001 compared to the INTACT group; ^##^*p* < 0.01 and ^###^*p* < 0.001 compared to the V-treated CCI group.

Significant upregulation of MMP-9 was detected in both the spinal cord (*p* < 0.001, **Figure 4A**) and DRG (*p* < 0.001, **Figure 4B**) in the V-treated CCI-exposed group comparing to INTACT group, and this elevation was prevented by repeated CORM-2 administration in both structures (in spinal cord: *p* < 0.001, **Figure 4A**, and in DRG *p* < 0.01, **Figure 4B**). TIMP-1 protein levels were not changed in either the V- nor CORM-2-treated CCI-exposed group (**Figures 4C,D**) comparing to INTACT group.

### Effects of Chronic Intrathecal CORM-2 Administration on the Protein Levels of Antinociceptive Factors in the Spinal Cord and DRG on Day 7 after CCI

**FIGURE 5:**
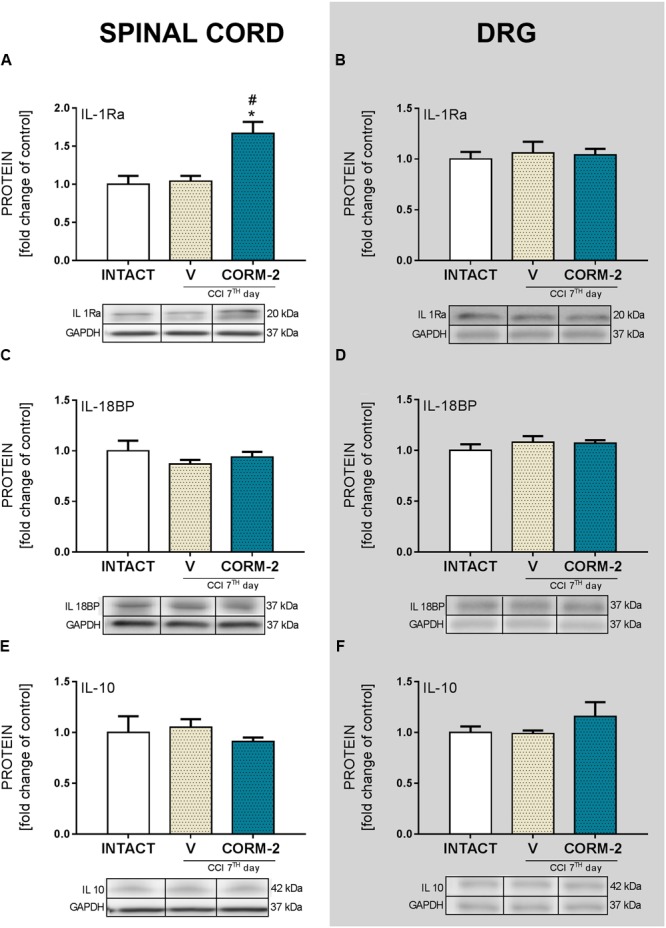
**The Western blot analysis shows the influence of repeated *ith.* CORM-2 administration on protein levels of IL-1Ra (A,B)**, IL-18BP **(C,D)**, and IL-10 **(E,F)** in the ipsilateral dorsal lumbar spinal cord **(A,C,E)** and dorsal root ganglia (DRG) **(B,D,F)** tissue on day 7 after chronic constriction injury (CCI) in rats 6 h after the last drug administration. The representative bands are shown below each column of respective group on the graph and come from the same membrane photo. Samples from different groups were not next to each other so were cut from different locations and set together. Data are presented as the mean ± SEM of 4–7 samples per group. Inter-group differences were analyzed using one-way ANOVA followed by Bonferroni’s multiple comparisons test. ^∗^*p* < 0.05 compared to the INTACT group; ^#^*p* < 0.05 compared to the V-treated CCI group.

CORM-2 (20 μg/5 μl, *ith.*) administration led to the elevation of antinociceptive IL-1Ra factor in the spinal cord (*p* < 0.05, **Figure 5A**), and its level in both the spinal cord and DRG of the V-treated CCI-exposed rats was not changed comparing to INTACT group (**Figures 5A,B**). We did not observe any changes in the levels of the other examined antinociceptive factors: IL-18BP (**Figures 5C,D**) or IL-10 (**Figures 5E,F**) after CCI or CORM-2-treatment comparing to INTACT group.

### Effects of Chronic Intrathecal CORM-2 Administration on p38MAPK, ERK1/2 and NFκB Protein Levels in the Spinal Cord and DRG on Day 7 after CCI

**FIGURE 6:**
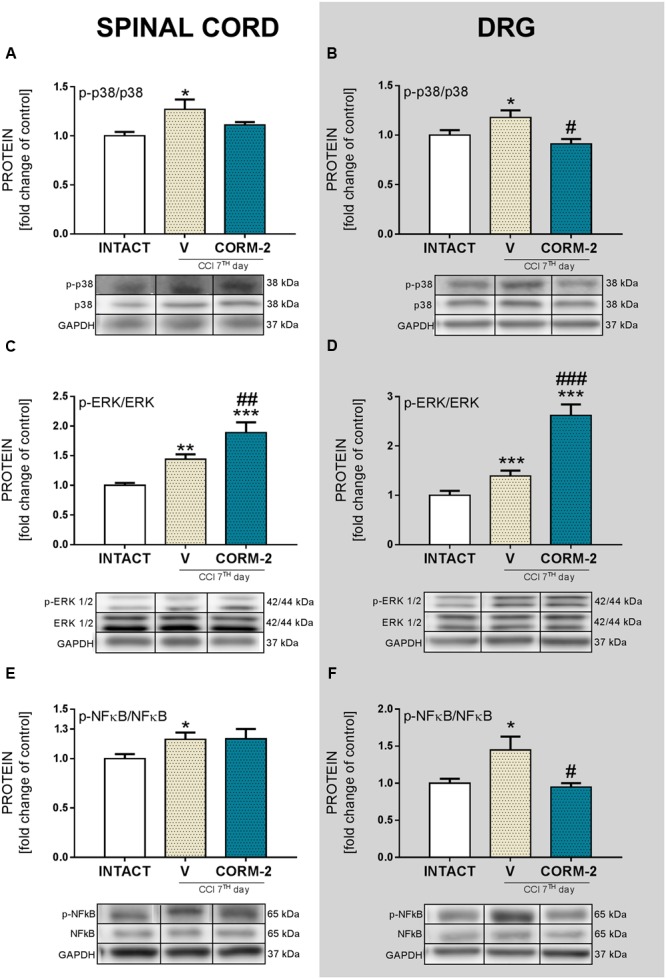
**The Western blot analysis shows the influence of repeated *ith.* CORM-2 administration on protein levels of p-p38 (A,B)**, p-ERK1/2 **(C,D)** and p-NFκB **(E,F)** in the ipsilateral dorsal lumbar spinal cord **(A,C,E)** and dorsal root ganglia (DRG) **(B,D,F)** tissue on day 7 after chronic constriction injury (CCI) in rats 6 h after the last drug administration. The representative bands are shown below each column of respective group on the graph and come from the same membrane photo. Samples from different groups were not next to each other so were cut from different locations and set together. Data are presented as the mean ± SEM of 4–7 samples per group. Inter-group differences were analyzed using one-way ANOVA followed by Bonferroni’s multiple comparisons test. ^∗^*p* < 0.05, ^∗∗^*p* < 0.01, and ^∗∗∗^*p* < 0.001 compared to the INTACT group; ^#^*p* < 0.05, ^##^*p* < 0.01, and ^###^*p* < 0.001 compared to the V-treated CCI group.

Repeated CORM-2 (20 μg/5 μl, *ith.*) administration prevented p38MAPK activation in the DRG (*p* < 0.05, **Figure 6B**), with insignificant attenuation in the spinal cord (**Figure 6A**) compared to that observed in V-treated CCI-exposed rats vs. INTACT group. ERK1/2 was significantly upregulated after CCI in spinal cord (*p* < 0.01, **Figure 6C**) and DRG (*p* < 0.001, **Figure 6D**) comparing to healthy animals. Moreover, CORM-2 treatment enhanced this elevation comparing to V-treated CCI-exposed rats also in both spinal cord (*p* < 0.01, **Figure 6C**) and DRG (*p* < 0.001, **Figure 6D**) NFκB levels of the CORM-2-treated group remained on similar elevated level as that of the V-treated CCI-exposed rats (*p* < 0.05, **Figure 6E**) when compared to that of the INTACT group in the spinal cord, and in the DRG, CORM-2 prevented the activation and upregulation of NFκB when compared to the vehicle treatment in the CCI-exposed rats (*p* < 0.05, **Figure 6F**).

### Effects of Chronic Intrathecal CORM-2 Administration on PI3K and Akt Protein Levels in the Spinal Cord and DRG on Day 7 after CCI

**FIGURE 7:**
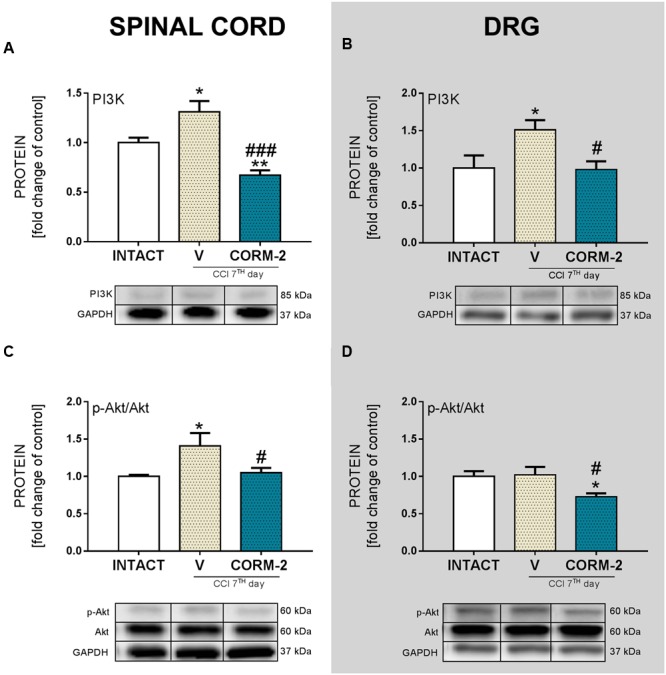
**The Western blot analysis shows the influence of repeated *ith.* CORM-2 administration on protein levels of PI3K (A,B)** and p-Akt **(C,D)** in the ipsilateral dorsal lumbar spinal cord **(A,C)** and dorsal root ganglia (DRG) **(B,D)** tissue on day 7 after chronic constriction injury (CCI) in rats 6 h after the last drug administration. The representative bands are shown below each column of respective group on the graph and come from the same membrane photo. Samples from different groups were not next to each other so were cut from different locations and set together. Data are presented as the mean ± SEM of 4–7 samples per group. Inter-group differences were analyzed using one-way ANOVA followed by Bonferroni’s multiple comparisons test. ^∗^*p* < 0.05 and ^∗∗^*p* < 0.01 compared to the INTACT group; ^#^*p* < 0.05 and ^###^*p* < 0.001 compared to the V-treated CCI group.

Repeated CORM-2 (20 μg/5 μl, *ith.*) administration prevented PI3K protein level upregulation in spinal cord (*p* < 0.001, **Figure 7A**) and DRG (*p* < 0.05, **Figure 7B**) comparing to V-treated CCI-exposed rats. Akt protein level was elevated in spinal cord (*p* < 0.05, **Figure 7C**) but not in DRG (**Figure 7D**). Those injections also prevented activation of Akt protein in spinal cord (*p* < 0.05, **Figure 7C**) and lowered, unchanged after CCI, its level in DRG comparing to V-treated CCI-exposed rats and INTACT group (*p* < 0.05, **Figure 7D**).

### Effect of a Single Intrathecal CORM-2 Administration on the Analgesic Effect of Morphine and Buprenorphine on Day 7 after CCI

**FIGURE 8:**
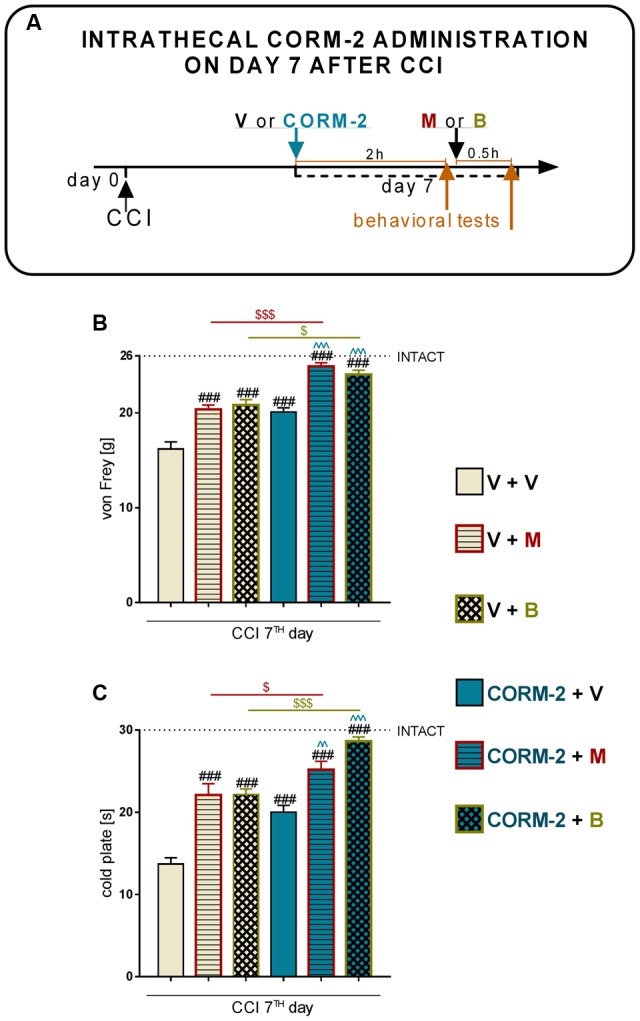
**Effects of a single *ith.* CORM-2 injection (20 μg/5 μl, *ith.*) on the (1) mechanical** (**B**; von Frey’s test) and thermal (**C**; cold plate test) hypersensitivity levels on day 7 after CCI as measured 2 h after drug administration (**A**; scheme), (2) analgesic effect of a single administration of morphine (M, 2.5 ug/5 μl, *ith.*) (**B,C;** V + M and CORM-2 + M), and (3) analgesic effect of a single administration of buprenorphine (**B**, 2.5 ug/5 μl, *ith.*) (**B,C**; V+ B and CORM-2 + B) as measured 0.5 h after opioids administration (**A**; scheme). The behavioral results are presented as the mean ± SEM (7–10 rats per group), and the horizontal dotted line shows the value of the INTACT group. The inter-group differences were analyzed using one-way ANOVA with Bonferroni’s multiple comparisons test. ^###^*p* < 0.001 compared to the V-treated CCI-exposed rats; *p* < 0.01 and *p* < 0.001 compared to the CORM-2-treated CCI-exposed rats; ^$^*p* < 0.05 and ^$$$^*p* < 0.001 between the V-CCI-opioid treated groups and CORM-2-opioid treated groups.

Single CORM-2 (20 μg/5 μl, *ith.*) administration had a similar analgesic effect to chronic administration, as measured 2 h after injection on day 7 after CCI with the von Frey’s test (*p* < 0.001, **Figure 8B**) and cold plate test (*p* < 0.001, **Figure 8C**). Single injection of both opioids produced analgesia in the V-treated CCI-exposed group in both tests (V-treated CCI-exposed group + morphine: *p* < 0.001, **Figures 8B,C**, V-treated CCI-exposed group + buprenorphine: *p* < 0.001, **Figure 8B**, C) 0.5 h after injection. Pretreatment with CORM-2, 2 h before treatment with opioids significantly enhanced their effectiveness in the von Frey’s (CORM-2-treated CCI-exposed group + morphine: *p* < 0.001, **Figure 8B**, CORM-2-treated CCI-exposed group + buprenorphine: *p* < 0.05, **Figure 8B**) and cold plate (CORM-2-treated CCI-exposed group + morphine: *p* < 0.05, **Figure 8C**, CORM-2-treated CCI-exposed group + buprenorphine: *p* < 0.001, **Figure 8C**) tests.

## Discussion

Our studies provide new evidence that spinal microglial P2X4R contribute to neuropathy development, since we observed spinal upregulation of P2X4R levels in every measured time point in parallel with activation of IBA-1-positive cells but no changes in the DRG. Here, we report that chronic, as well as single pharmacological blockade of P2XR with CORM-2 (*ith.* administration, once daily for 9 days) diminished both mechanical and thermal hypersensitivity in a rat model of neuropathic pain on day 7 after CCI, which is a continuation of our previously published results suggesting an important role of P2X4R, however, we have measured only its influence on thermal hypersensitivity at that time (Jurga et al., 2016a). Our results indicate, for the first time, that blockade of P2X4R with chronic *ith.* administration of CORM-2 prevents elevated levels (on day 7 after CCI) of glial markers (IBA-1 and GFAP) in the spinal cord and/or DRG bringing it closer to the level observed in the INTACT animals. A certain amount of pronociceptive factors, e.g., cytokines, are known to be upregulated in neuropathic pain and in the CCI model of neuropathy. We have examined the protein levels of IL-1β, IL-18, and IL-6 released by activated glial cells in the spinal cord and DRG. The results we report suggest that blockade of microglial P2X4R transmission brings those factors back to physiological levels. The antinociceptive factors we examined (IL-1Ra, IL-18BP, and IL-10) remained at the same level after CCI as those in INTACT animals; however, we observed a strong upregulation of IL-1Ra in CORM-2-treated CCI-exposed rats. The observation that even a single *ith.* administration of CORM-2 has additive analgesic with morphine and buprenorphine on day 7 after CCI supplements our previous results on chronic *ip.* administration of CORM-2 and suggests that P2X4R is an interesting molecular target for the treatment of neuropathic pain.

As we have reported previously (Jurga et al., 2016a), activated spinal microglia upregulation starts as early as day 2 after CCI, with the biggest change on day 7, and lasts until day 14, when it is still significant. Similarly, upregulation of microglia protein markers was reported by others in the rat and mouse sciatic nerve injury model on day 7 (Bishay et al., 2010) and day 14 (Kang et al., 2015). Astrocyte upregulation was reported in CD1 mice in the CCI model on days 10 and 21 after surgery (Chen et al., 2014), which, in combination with our results, indicate that astroglia are activated later than microglia. In relation to those results, we have observed spinal upregulation of the P2X4R protein level parallel to microglial activation. This suggests a relation between P2X4R and microglial activation in neuropathy development in the Wistar rat CCI model, especially considering that P2X4R was reported to co-localize only with microglia and not with astrocytes or neurons in the central nervous system (Tsuda et al., 2003). Results from the Wistar rat SNL model showed upregulation in P2X4R protein levels on days 1, 3, 7, and 14 (Tsuda et al., 2003) and in the Sprague-Dawley rat CCI model on days 3, 7, and 14 (Chen X.M. et al., 2015). DRG tissue analysis has not been reported yet; however, here we show that the P2X4R level remains unchanged. This supports the hypothesis on the contribution of microglial P2X4R to neuropathy development because IBA-1-positive cells responsible for the upregulation in the DRG are macrophages and not microglia.

ATP, acting via P2 purinergic receptors, has been suggested to mediate neuropathic pain development. The ATP-gated P2X4R is considered to be involved in the modulation of spinal microglia activity and peripheral macrophages and in the maintenance of hypersensitivity caused by peripheral nerve injury (Trang and Salter, 2012). Here, we report that pharmacological blockade of P2X4R with CORM-2 (repeated and single *ith.* administration) has the potential to diminish both mechanical and thermal hypersensitivity in the rat CCI model of neuropathic pain as measured on day 7 after the operation. This complements our previously published results regarding *ip.* administration (Jurga et al., 2016a) and supports the hypothesis on the important role of P2X4R in neuropathic pain development. Moreover, treatment with other available P2XR antagonists showed results also favoring this supposition. For instance, experiments on mice where the effects of two antagonists of P2XR were compared (one selective to P2X4R and one to all other P2XRs) clearly indicated that P2X4R is the most important among the P2X receptor family (Tsuda et al., 2003). Until now, it has been reported that *ip.* administration of CORM-2 has an analgesic potency in a mouse SNL model (Liu et al., 2015), a mouse inflammatory pain CFA model (Negrete et al., 2014) and in a Sprague-Dawley rat CCI model 14 days after CCI (Chen Y. et al., 2015). Blockade of receptors on microglia cells is a promising target for neuropathic pain pharmacotherapy, as reports regarding antagonism of Toll-like receptors 4 (Lewis et al., 2013; Jurga et al., 2016b), histamine H4 receptors (Medhurst et al., 2008; Hsieh et al., 2010; Dong et al., 2014), or chemokine receptors (e.g., CCR5, CXCR4) (Bhangoo et al., 2007; Hu et al., 2015; Bai et al., 2016; Kwiatkowski et al., 2016; Luo et al., 2016; Piotrowska et al., 2016) are increasingly common. We decided to investigate the changes caused by P2X4R antagonism with CORM-2 (repeated *ith.* administration) in some pathways related to the activity of those receptors whose role in neuropathic pain has already been proposed. First, we examined the p38MAPK pathway. It is known that p38MAPK is activated in microglia/macrophages cells in neuropathic pain models (Jin et al., 2003; Tsuda et al., 2004). Interestingly, it is reported that TNP-ATP, a specific P2X4R antagonist, has the ability to downregulate this receptor in microglial cultures when its level was previously elevated after ATP stimulation (Tsuda et al., 2003). This draws attention to the possible relationship between activation of P2X4R and the p38MAPK pathway, which leads to the enhanced production of pronociceptive factors, including NO and cytokines (DeLeo and Yezierski, 2001), and to the activation of transcription factors responsible for regulation of genes involved in nociception (Potucek et al., 2006). Pharmacological inhibition of p38MAPK and activation of microglia/macrophages has analgesic potency in neuropathic pain models, as has been reported with minocycline (Tikka et al., 2001; Popiolek-Barczyk et al., 2015) or SB203580 (Tsuda et al., 2004) treatment. In our experiments, we observed a downregulation of p38MAPK by CORM-2 in the DRG but not in the spinal cord in the neuropathic pain model. Another member of the MAPK family that we examined was ERK1/2, which is strongly activated within neurons and glia during neuropathic pain. Our results are in agreement with previously published results that show that ERK1/2 phosphorylation is increased on day 7 after CCI (Rojewska et al., 2015), which is correlated with enhanced glial cell activation (Popiolek-Barczyk et al., 2015). Previously, published results suggested that ERK1/2 is essential for the intracellular signaling that leads to the production of various nociceptive factors, including cytokines, that participate in the intensification of the neuropathic pain sensations (Popiolek-Barczyk et al., 2015; Rojewska et al., 2015). In our experiments, we measured the spinal upregulation of p-ERK1/2 by CORM-2 in the neuropathic pain model and, in parallel, the level of antinociceptive IL-1Ra. However, whether this or other (unknown yet) relationship is important for the observed effect requires clarification and further studies. In the DRG we did not observe any impact of CORM-2 administration on IL-1Ra levels, which can be explain by *ith.* administration. Excessive production of the above-mentioned pronociceptive factors, e.g., interleukins, are known to be upregulated in neuropathy models (Verri et al., 2007; Rojewska et al., 2014, 2015; Popiolek-Barczyk et al., 2015). IL-1β and IL-18 promotes pain behavior development when injected intrathecally (Malcangio et al., 1996; Miyoshi et al., 2008). IL-1β, IL-18 and IL-6 are spinally upregulated in neuropathic pain models, including our reports (Okamoto et al., 2001; Pilat et al., 2015, 2016; Rojewska et al., 2016). Interestingly, the results we observed suggest that blockade of microglial P2X4R transmission restores the elevated levels of pronociceptive factors in neuropathy. Moreover, we have shown that the MMP-9 level is elevated in rats after CCI in the spinal cord and DRG (Schonbeck et al., 1998; Kawasaki et al., 2008; Rojewska et al., 2014), but here, we are the first to show that preemptive and repeated P2X4R blockade has the ability to prevent this upregulation. This observation is important, since Kawasaki et al. (2008) has proven that MMP-9 produced in the injured DRG neurons serves as one of the triggers for spinal microglia activation and neuropathic pain development and that MMP-9-induced pathophysiology involves IL-1β cleavage and microglia p38MAPK activation. These observations, along with the identical regulation of pronociceptive spinal IL-1β in the spinal cord, are extremely important. Our results are also in agreement with Vaccari et al. (2012), who reported a decrease in IL-1β release in P2X4R^-/-^ mice after spinal cord injury, which resulted in an improvement in the functional outcome. The results of our studies provided evidence that blocking P2X4Rs prevents microglial activation and, consequently, the release of pronociceptive molecules. It is claimed that under neuropathic pain, the physiological balance between pronociceptive and antinociceptive factors is disturbed (Mika et al., 2008; Rojewska et al., 2014, 2015). The corresponding antinociceptive factors that we examined were expressed at the same level as those in the INTACT group on day 7 after CCI. However, we observed a strong upregulation of spinal IL-1Ra in CORM-2-treated CCI-exposed rats. IL-1Ra is a member of the IL-1 family that binds to IL-1 receptors but does not induce any intracellular response. The IL-1Ra blocks the pronociceptive effects of IL-1β and therefore has a strong analgesic effect under neuropathy (Hook et al., 2011; Pilat et al., 2015). Chronic *ith.* administration of CORM-2 inhibited microglial activation in neuropathy and enhanced the level of IL-1Ra, an important antinociceptive factor. We also examined the level of IL-18BP, which is known to inactivate pronociceptive IL-18 (Kim et al., 2000; Pilat et al., 2016); however CORM-2 administration did not influence its level. Another important antinociceptive cytokine is IL-10 (Bluthé et al., 1999; Heyen et al., 2000), but like IL-18BP, its level was not regulated by CORM-2 administration. This may suggest that the mechanism of IL-1Ra activation relies on the P2X4R-mediated pathway and that this molecule, along with pronociceptive IL-1β (IL-1Ra blocks its activation), plays a key role in neuropathy development.

ATP, an endogenous agonist of purinergic receptors, has the ability to activate p38MAPK with the release of cytokines and other microglial factors engaged in nociception. According to the literature, the second pathway important to investigate was the NLRP3/Caspase-1 inflammasome, because this complex is responsible for the cleavage of the preforms of IL-1β and IL-18 into active molecules (Rathinam et al., 2012). Moreover, Jiang L. et al. (2016) reported that in mice, CORM-2 administration in an acute lung injury model downregulates the level of elevated NLRP3/Caspase-1 in the lung bronchoalveolar lavage fluid, suggesting engagement of P2X4R in the activation of the inflammasome. However, we did not observe activation of this complex in the CCI model, which we found was in agreement with the studies of Curto-Reyes et al. (2015) who recently reported that pain development is not dependent on NLRP3/Caspase-1 in a mouse SNL model (**Table 1**).

**Table 1:** The Western blot analysis showed the influence of repeated *ith.* CORM-2 administration on protein levels of Nucleotide-binding oligomerization domain, Leucine-rich Repeat and Pyrin domain containing 3 (NLRP3) and Caspase-1 (Casp-1) in the ipsilateral dorsal lumbar spinal cord and dorsal root ganglia (DRG) tissue on day 7 after chronic constriction injury (CCI) in rats 6 h after the last *ith.* CORM-2 administration.

	Spinal cord			DRG		
	INTACT	V CCI	CORM-2 CCI	INTACT	V CCI	CORM-2 CCI
NLRP3	1.00 ± 0.03	1.00 ± 0.11	0.96 ± 0.07	1.00 ± 0.06	1.16 ± 0.01	1.15 ± 0.05
Casp-1	1.00 ± 0.03	1.19 ± 0.10	1.18 ± 0.11	1.00 ± 0.03	0.94 ± 0.04	0.97 ± 0.06

*Data are presented as the mean ± SEM of 4–7 samples per group. Inter-group differences were analyzed using one-way ANOVA followed by Bonferroni’s multiple comparisons test.*

The recent literature data suggest that activation of PI3K/Akt pathway is strongly involved in release of interleukins and other microglial factors involved in nociception, which is in agreement with our results showing the activation of PI3K/Akt pathway after CCI in rats on day 7. Similarly, it has been presented, that this pathway is upregulated undergo activation, e.g., in Oxaliplatin chemotherapy-induced neuropathic pain in mice (Jiang S.P. et al., 2016). Significant upregulation of p-Akt in DRG neurons was also reported after L5 spinal nerve ligation rat model, with attenuation of behavioral pain symptoms after PI3K inhibitors administration (wortmannin or LY294002) (Xu et al., 2014). It was proven that AS605240 (PI3K inhibitor) produced a significant relief in tripsine-induced nociception in mice (Pereira et al., 2011). Not to neglect remains also the fact, that administration of PI3K inhibitor (LY294002) into primary microglial culture suppresses the P2X4R upregulation after fibronectin introduction (Tsuda et al., 2009). Moreover, it has been shown, using chemoattractant protein-1 (MCP1)-mediated bone cancer pain model, that there is a significant dependence between PI3K/Akt and microglia activation (Jin et al., 2015). Here we show that repeated *ith.* administration of CORM-2 lowers levels of both PI3K and p-Akt protein in spinal cord and DRG suggesting its significant contribution to P2X4R-mediated pain development.

The P2X4R antagonist has potency to not only diminish mechanical and thermal hypersensitivity in a rat CCI model of neuropathic pain but also enhance opioid analgesia. The observation that a single *ith.* administration of CORM-2 enhances the analgesic effects of single-injected morphine and buprenorphine on day 7 after CCI supplements our previous results obtained after *ip.* administration of CORM-2 (Jurga et al., 2016a). It is known that opioids, commonly used in the clinic for inflammatory or acute pain therapy, lose their efficacy in neuropathic pain. This may be connected with the development of tolerance (Narita et al., 2008), downregulation of opioid receptors (Mika et al., 2014) and activation of p38MAPK along with the enhanced release of pronociceptive factors (Raghavendra et al., 2004; Hutchinson et al., 2008a). Inhibition of microglia activation and secretion of nociceptive factors by certain pharmacological tools has been shown to improve opioid analgesia, for example treatment with an inhibitor of microglia – minocycline (Hutchinson et al., 2008b), pronociceptive interleukin – propentofylline (Miki and Miki, 1991; Raghavendra et al., 2004) or p38MAPK inhibitor - SB203580 (Cui et al., 2006). Moreover, in 2015 authors suggested that the activation of PI3K/Akt pathway can influence the opioid effectiveness, which stays in agreement with our pharmacological results showing both morphine and buprenorphine analgesic properties are enhanced by CORM-2, inhibitor of this pathway (Lutz et al., 2015). According to those results, pharmacological blockade of P2X4R may be a promising target for neuropathic pain therapy because, as we report here, CORM-2 administration leads to the attenuation of pronociceptive cytokine levels and significant analgesia with the ability to enhance both morphine and buprenorphine effectiveness. Together, this indicates that P2X4R may play an important role in nociception and opioid effectiveness in neuropathic pain and is, therefore, a promising target for effective therapy construction.

## Conclusion

**SCHEME 1:**
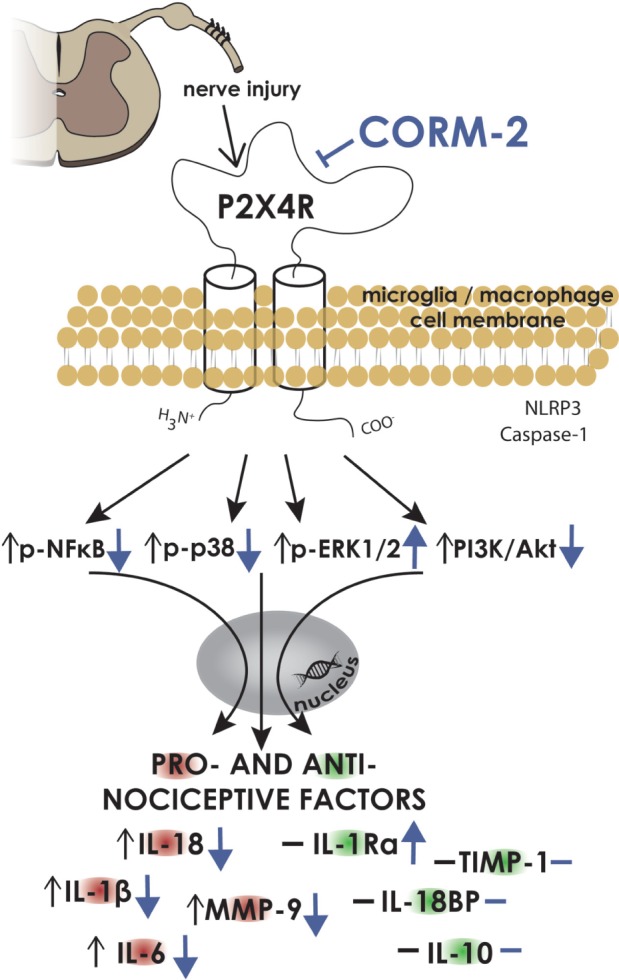
**CORM-2 influences IBA-1- and GFAP-positive cells and many nociceptive factors and signaling pathways as measured on day 7 after CCI.** Our data strongly support the hypothesis that P2X4 receptors play a significant role in neuropathy. In the nervous system, they are localized on microglia/macrophages (Tsuda et al., 2003), and we have observed that the elevation of levels of IBA-1- and GFAP-positive cells in the spinal cord and DRG after chronic constriction injury (CCI) is prevented by preemptive repeated administration of the P2X4 antagonist – CORM-2. Additionally, pronociceptive factors released by those cells in neuropathy are regulated by CORM-2 in a similar manner (IL-1β, IL-18, IL-6, MMP-9) in the spinal cord and/or DRG. Additionally, P2X4R blockade also has the ability to enhance production of spinal antinociceptive factor, IL-1Ra. We already know from experiments using minocycline that the microglial p38MAPK pathway is engaged in neuropathic pain (Jin et al., 2003), and here, we have shown for the first time that P2X4R signaling also influences this pathway by preventing the upregulation of p38 and enhancing ERK1/2 in the spinal cord and/or DRG. In contrast, we did not detect any change in the NLRP3/Casp-1 inflammasome complex, which is in agreement with the literature (Curto-Reyes et al., 2015). In summary, pharmacological blockade of P2X4 receptors diminished pain and increased the effectiveness of opioids in neuropathy by influencing a variety of cell signaling pathways that are implicated in nociception. The P2X4 receptor can be a potential target for a new and more successful treatment for neuropathic pain. CORM-2,CO releasing molecule 2; DRG, dorsal root ganglia; P2X4R, P2X4 receptor; IBA-1, ionized calcium-binding adapter molecule 1; GFAP, glial fibrillary acidicprotein; IL, interleukin; IL-1Ra, interleukin 1 receptor antagonist;IL-18BP, interleukin 18 binding protein; p38, p38 mitogen-activated protein kinase; ERK1/2, extracellular signal-regulated kinase ½; NFκB, nuclear factor-kappa β; NLRP3, nucleotide-binding oligomerization domain, leucine rich repeat and pyrin domain containing 3; Casp-1, caspase 1; MMP-9, matrix metalloproteinase 9; TIMP, tissue inhibitor of metalloproteinases. Graphical data are presented as changes in the V-treated CCI-exposedrat group compared to the INTACT group (black arrows or lines) and the CORM-2-treated CCI-exposed rat group compared to the V-treated CCI-exposed rat group (blue arrows or lines). The upright position of thearrow symbolizes the upregulation of the corresponding factor, and the downward position symbolizes its downregulation. The horizontal line symbolizes no change between the above-mentioned groups.

Our results support the hypothesis that spinal microglial P2X4R contribute to neuropathy development, since we have observed spinal upregulation of P2X4R levels at every time point in parallel with activation of IBA-1-positive and GFAP-positive cells. Our results confirmed that preemptive and repeated pharmacological spinal blockade of P2X4R can attenuate pain as well as improve opioid analgesia on day 7 after CCI. Interestingly, we observed (**Scheme 1**) that CORM-2 inhibited pronociceptive factors (IL-1β, IL-18, IL-6) in the spinal cord and/or DRG along with the MMP-9 factor, which is a well-known contributor to IL-1β activation and spinal microglia activation. Moreover, CORM-2 treatment led to the elevation of antinociceptive IL-1Ra, a molecule whose action opposes that of pronociceptive IL-1β. With strong support from our results summarized in **Scheme 1**, we are proposing a hypothesis that MMP-9, p38MAPK, ERK1/2 and PI3K/Akt, not TIMP-1 and the NLRP3/Caspase-1 inflammasome complex, are involved in the analgesic effects of CORM-2 in rat neuropathic pain. Taking this into consideration, it is highly probable that microglial P2X4R may be involved in nerve-injury caused nociception because our results showed that treatment with CORM-2 restores the physiological level of pronociceptive factors in neuropathy and attenuates pain. This suggests that P2XR is a promising target for pharmacomodulation in future trials.

## Ethics Statement

Only the minimal essential number of animals was used, and all of the procedures were performed according to the recommendations of IASP (Zimmermann, 1983) and the NIH Guide for the Care and Use of Laboratory Animals. This study was carried out in accordance with the recommendations of local Ethics Committee (Krakow, Poland), permission number: 1055.

## Author Contributions

AJ, AP, WM, BP, and JM performed the experiments, AJ and JM designed the study, AJ, AP, JM analyzed and interpreted the results, AJ, AP, WM, BP, and JM drafted the manuscript and accepted the finalized version.

## Conflict of Interest Statement

The authors declare that the research was conducted in the absence of any commercial or financial relationships that could be construed as a potential conflict of interest.

The reviewer LVD and handling Editor declared their shared affiliation, and the handling Editor states that the process nevertheless met the standards of a fair and objective review.
